# An Uncommon Ventricular Tachycardia due to Inactive PPM Lead

**DOI:** 10.5402/2011/232648

**Published:** 2011-04-14

**Authors:** Goutam Datta, A. Sarkar, A. Haque

**Affiliations:** Division of Cardiology, Institute of Postgraduate Medical Education and Research, Kolkata 700020, India

## Abstract

Our patient had recurrent syncope due to ventricular tachycardia (VT) after one year of VVI Pacemaker implantation. He had pacemaker pocket infection for which new pacemaker was implanted on opposite side but old lead was not explanted completely. Flouroscopy showed redundant loop of old lead in right ventricular inflow which was snared out subsequently. He never had syncope or VT after that.

## 1. Case Report

A sixty-year-old male presented to emergency with recurrent episodes of syncope for the last two weeks. He had VVI. pacemaker implantation one year ago in another centre. Subsequently, he had developed pacemaker pocket infection for which pulse generator was removed in the same centre and reimplanted in the contralateral side two weeks prior to presentation. At that time, old lead could not be removed fully and a new lead was used in the opposite side. 

This time, according to the patient, syncope occurred in reclining position at particular angle but never during supine or standing position. And during presyncope, if he or his relatives changed the position, syncope averted. This was subsequently corroborated by us in the hospital. His biochemical parameters were within normal limits.

**Figure 1:**
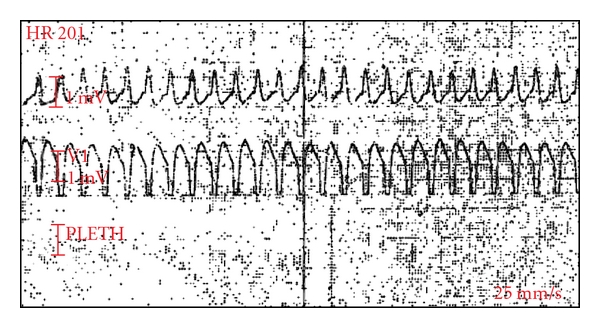
Ventricular tachycardia from right ventricle.

Echocardiography revealed normal biventricular function. Coronary angiography was normal. Fluoroscopic examination showed redundant loop of old lead in right ventricular inflow. Our impression was that the redundant loop might have been stimulating right ventricle causing ventricular tachycardia in particular position. We have documented ventricular tachycardia during hospital stay in particular position and immediate resumption of either sinus or pacemaker-dependent rhythm on changing the position of the patient (Figure 1).

**Figure 2:**
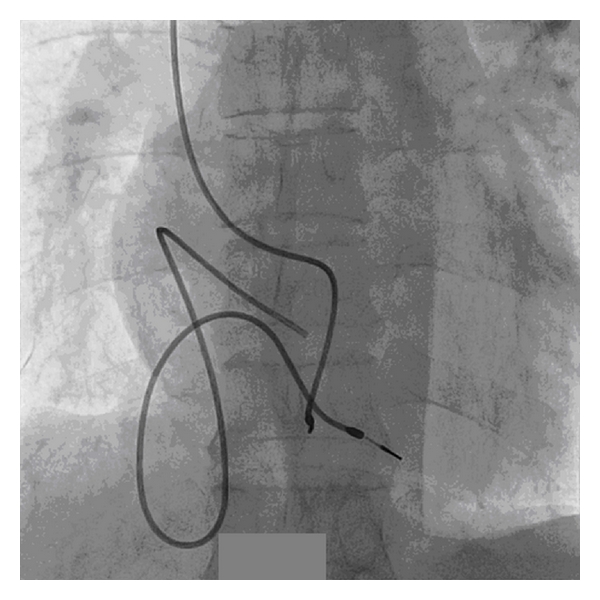
Redundant loop of old lead and new pacemaker lead from opposite side.

**Figure 3:**
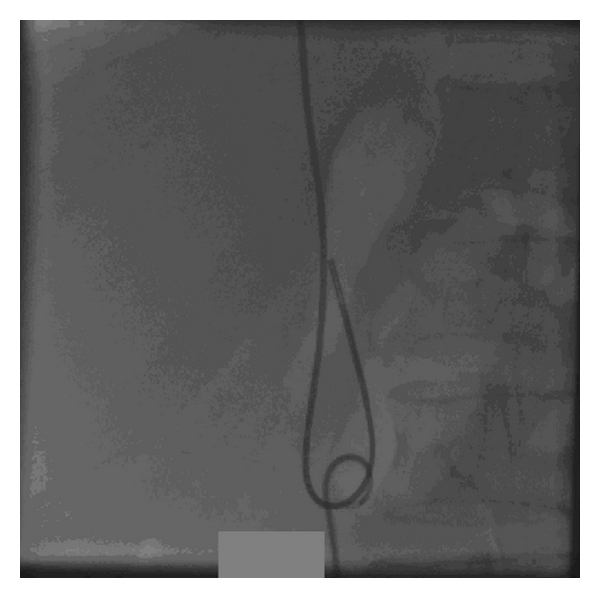
Old pacemaker lead is straightened by pigtail catheter.

**Figure 4:**
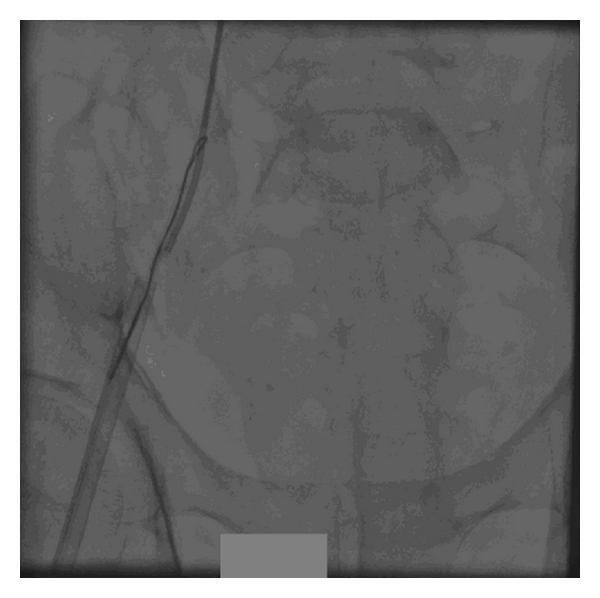
Redundant loop is snared out.

With this idea, we explanted the old lead through transfemoral route. Redundant loop of old lead was initially straightened by pigtail catheter. After that, it was snared out by gentle traction through 12 F femoral venous sheath (Figures 2, 3, and 4).

After that patient never had VT during two months of followup. This is an interesting case where redundant loop of lead was responsible for ventricular tachycardia.

## 2. Discussion

 Syncope following permanent pacemaker implantation is a nightmare for electrophysiologists. VT may occur after pacemaker implantation for several reasons. Lindsay et al. reported a case of ventricular tachycardia four months after dual chamber pacemaker implantation [1]. Patient's right ventricular pacing wire had wrapped around tricuspid annulus. After repositioning, patient did not have syncope or VT. Bohm et al. presented another case of symptomatic nonsustained ventricular tachycardia induced by mechanical irritation of the right ventricular outflow tract [2]. Repositioning of pacemaker loop eliminated the rhythm disturbance. Lefroy et al. reported a case of ventricular tachycardia following VVI pacemaker implantation due to fusion of a ventricular extrasystole with a pacemaker beat [3], whereas pacemaker stimulus on T wave was reason for VT in the case described by Freedman et al. [4]. Reentry circuit around endocardial pacemaker lead may also be a reason for VT in a patient with pacemaker [5]. Iesaka et el. reported a case of bradycardia-dependent VT which was facilitated by long pause caused by myopotential inhibition of a VVI pacemaker [6]. Lead-lead interactions may occur in presence of multiple leads. Ventricular tachycardia and sudden cardiac death occur in 12–31% of patients following permanent pacemaker implantation [7]. Coronary artery disease and left ventricular dysfunction account for majority of these cases. In our patient coronary angiography was normal with good biventricular systolic function. Recently, one case of proarrhythmia caused by ICD lead was also reported, which was cured by lead extraction [8]. Under the circumstances in which VT occurred in our patient, it may be inferred that redundant loop was responsible for VT and syncope in our patient. This is an interesting case implicating abandoned PPM lead as a cause of VT where positional variation of the loop of abandoned PPM lead resulted in a positional-dependant VT and syncope. 

##  Conflict of Interests

The author declared no conflict of interests.
